# Anisotropic Thermal Conduction in Transition Metal Dichalcogenide Nanocomposites with Rough Interfaces

**DOI:** 10.3390/nano8121054

**Published:** 2018-12-15

**Authors:** Iorwerth O. Thomas, Gyaneshwar P. Srivastava

**Affiliations:** School of Physics, University of Exeter, Stocker Road, Exeter EX4 4QL, UK; G.P.Srivastava@exeter.ac.uk

**Keywords:** effective medium approach, thermal conductivity, anisotropic systems, nanocomposites

## Abstract

We present a theory of thermal conduction in a transition metal dichalcogenide nanocomposite structure with rough interfaces that accounts for the anisotropic conductivities of the host, the insert and the interface regions. The host and insert conductivities are calculated using a semi ab-initio method. The effects of specularity in phonon interface scattering and the thermal boundary resistance is incorporated through linking a phonon wavevector dependent specular scattering parameter to the average height of surface inhomogeneities, and the conductivity of the composite is calculated by employing an extension of a modified effective medium approach. Our work for spherical inserts of WS${}_{2}$ in MoS${}_{2}$ predicts that the effects of specular scattering due to surface roughness is more pronounced for inserts smaller than 100 nm, even at volume fractions of the order of 0.05.

## 1. Introduction

The determination of key parameters of a nanocomposite structure that control its physical properties, such as thermal, electronic and optical characteristics, is essential for future technological advances. The calculation of the bulk properties of nanocomposites where a small fraction of insertions of one material are embedded within a matrix of a different material, perhaps with a boundary layer between the insertion and the matrix, is a long-standing problem. Effective medium approaches (EMAs) are a widely used method of calculating properties (such as the thermal conductivity [1] or the dielectric constant [2]) of composite systems using the properties of their individual constituents [2,3]. In thermal or electrical conductivity calculations, these constituents usually consist of inserts of one material embedded in a matrix of another, along with the effects of the boundary between them. Such a boundary is usually considered to be either an infinitely thin interface layer possesed of a Kapitza or interface resistance [4] (e.g., Ref. [1]) or a finite region with a conductivity of its own (e.g., Ref. [5]). The former approach can be adapted to model a wide variety of systems, for instance cases involving a bimodal distribution of insert particle sizes (e.g., Ref [6]) or systems containing pores (e.g., Ref [7]). However, this form of EMA is only valid for micrometer sized inserts, and requires further modification if it is to be valid for nanocomposite systems. For thermal conductivity calculations, Minnich and Chen [8] proposed a modified effective medum approach (mEMA) which is suitable for nanoscale systems such as the recently synthesised Bi${}_{100-x}$Sb${}_{x}$/Al${}_{2}$O${}_{3}$ nanocomposites [9]. In the mEMA the effects of phonon scattering from the boundaries of spherical inserts are included in the calculation of the matrix and insert thermal conductivities that are input into the closed-form expression for the effective thermal conductivity. This approach has been generalised to account for different insert shapes, where the effects of boundary scattering are not isotropic [10,11,12,13], and also to systems containing multiple particle sizes and orientations [14].

In composites formed of anisotropic materials (e.g., layered systems such as transition metal dichalcogenides) the directional dependence of the matrix thermal conductivity or dielectric constant must be accounted for [15,16,17]. If a Kapitza resistance exists in such a system, it is reasonable to assume that will also be directionally dependent and must be accounted for in a similar fashion (the principle of doing so within a Morika-Tanaki approach has been discussed in, for example, Ref. [18]). Within the context of the multiple scattering approach used in this study, an extension to Minnich and Chen’s [8] approach that accounts for anisotropic boundary resistance effects (emEMA) has been recently proposed in [19].

However, the originally formulated mEMA [8] and its recent extension [19] assume that the phonon scattering from insert boundaries is fully diffusive. In other words, these formulations treat the boundary as being perfectly rough. This is unlikely to be the case in real systems, since fully diffusive and specular scattering are idealised limiting cases. A simple phenomenological approach to the problem has been proposed [11], in which an empirically weighted average of the two limits is calculated. While this gives physically plausible results, there is no obvious connection between the value of the weighting parameter and the physical properties of the nanocomposite. In this work we suggest a scheme based upon Koh et al. [20] proposed correspondence between the relative proportions of different boundary scattering types and the average height of inhomogeneities on the boundary layer that is wave-vector dependent. In other words, our proposed extenstion improves on the approach adopted by Behrang et al. [11] for including both specular and diffuse scattering contributions to phonon boundary scattering rate as well as the thermal interface (Kapitza) scattering term.

Transition metal dichalcogenides (TMDCs) such as MoS${}_{2}$ and WS${}_{2}$ possess high electrical conductivities and low thermal conductivities. Because of this, they are thought to be good candidates for use in thermoelectric applications. Recent studies [21,22,23] demonstrate early attempts at fabricating TMDC-based nanostructures and composites. Through a judicious choice of insert and matrix material it will be possible to further reduce the thermal conductivity of these systems without strongly affecting the thermoelectric power factor, and so improve the thermoelectric figure of merit. In order to accurately predict the thermal conductivity reduction in a given nanocomposite, some means of accounting for the quality of the matrix-insert interface is required. Therefore in anticipation of future thermal conductivity measurements, we implement this scheme within the emEMA for a layered (hence anisotropic) bulk transition metal dichalcogenide nanocomposite consisting of spherical inserts of bulk 2H WS${}_{2}$ embedded within a matrix of bulk 2H MoS${}_{2}$, where semi-ab initio calculations [24] are used to obtain the required insert and matrix thermal conductivities.

## 2. Theoretical Method

An emEMA calculation for a system with anisotropic interface resistance is performed in two steps. Firstly we compute the effective thermal conductivity of the insert and the interface regions [19]:

(1) $$\kappa^{*}=\kappa^{i}{\left[I+\frac{3}{a}N_{s}\kappa^{i}R^{K}\right]}^{-1},$$

where $\kappa^{i}$ is the thermal conductivity of the insert in the absence of the boundary, $\kappa^{*}$ is the effective thermal conductivity of the insert and the boundary, *a* is the radius of the spherical insert, $R^{K}$ is the thermal boundary or Kapitza resistance, and $N_{s}$ is the surface depolarisation tensor (see Appendix A) accounting for the effects of anisotropic Kapitza resistance. The thermal conductivities, depolarisation tensor, Kapitza resistance and identity matrix I are 3×3 matrices, and we assume that the system is oriented so that $\kappa^{i}$ and $R^{K}$ are diagonal.

We then calculate the thermal conductivity of the effective medium $\kappa^{E}$ using the standard anisotropic EMA equation [15]:

(2) $$\begin{array}{cc} \kappa^{E} & =\kappa^{m}+f\left(\kappa^{*}-\kappa^{m}\right)J^{-1}\kappa^{m},\\ J & =\kappa^{m}+\left(1-f\right)N_{m}\left(\kappa^{*}-\kappa^{m}\right). \end{array}$$

Here *f* is the volume fraction of inserts, $\kappa^{m}$ is the thermal conductivity of the matrix and $N_{m}$ the matrix depolarisation tensor (see Appendix A) accounting for any anisotropy in $\kappa^{m}$. We assume the simplest possible case where $\kappa^{m}$ and $\kappa^{*}$ are both diagonal.

The required values of $\kappa^{i}$ and $\kappa^{m}$ may be computed via any reliable method for calculating the thermal conductivity. In this work we use a semi-ab initio method [24], which we summarise in Appendix B.

In order to include the effects of specularity, we define a momentum dependent parameter $s_{q}$ as the fraction of modes of momentum q that undergo diffusive scattering from the insert interfaces. Generalising the simple expression of Koh et al. [20], we write:

(3) $$s_{q}=1-l_{q},$$

where $l_{q}=\exp\left(-\epsilon^{2}/\lambda_{q}^{2}\right)$ with ϵ being the average height of surface inhomogeneities and $\lambda_{q}$ being the wavelength corresponding to momentum q. We define $\epsilon =\eta a_{0}$ where $a_{0}$ is the lattice spacing, and $\lambda_{q}=2\pi /Q$, where $Q=2\pi |q|/a_{0}$ and $\left|q\right|=\sqrt{q_{x}^{2}+q_{y}^{2}+q_{z}^{2}}$ is the norm of the lattice momentum vector in Cartesian co-ordinates. From these definitions it follows that:

(4) $$s_{q}=\left(1-e^{-\eta^{2}{\left|q\right|}^{2}}\right).$$

The value of η controls the extent of specular scattering. If η→0, then the surface becomes smooth and we obtain the specular limit $s_{q}=0$. If η→∞ then the surface becomes infinitely rough and we obtain the diffuse limit $s_{q}=1$. Physically meaningful values of η will lie somewhere between these extremes, although in principle it is possible for a surface to be smooth enough or rough enough that all scattering is effectively specular or diffusive.

The effects of finite η enter into our calculation on two levels: through the thermal boundary resistance at the EMA level, and through modifications of the effective boundary lengths of the matrix and insert at the mEMA level. We begin with the latter.

Scattering from the sample boundary itself is taken to be purely diffusive and unaffected by the specularity parameter. Minnich and Chen [8] include the effects of phonon scattering from insert interfaces by means of effective boundary lengths, and these must be modified so as to include the effects of specularity. In the case of the insert, any mode not scattered from the interface boundary due to specularity must be scattered from the sample boundary, and so the effective boundary length must be a weighted average of the sample length and the insert diameter. In the case of the matrix, only the interface density contribution to the effective boundary length is affected by the specularity. We may therefore write the the effective boundary length $L_{B}$ as follows:

(5) $$L_{B}^{-1}=\left\{\begin{array}{cc} s_{q}L_{B,\;I}^{-1}+l_{q}L_{B,\;S}^{-1} & \;\mathrm{for}\;\mathrm{inserts}\;\\ L_{B,\;S}^{-1}+s_{q}L_{IS}^{-1} & \;\mathrm{for}\;\mathrm{the}\;\mathrm{matrix} \end{array}\right.,$$

where $L_{B,\;I}=2a$ is the insert boundary length, $L_{B,\;S}$ is the sample boundary length, $L_{IS}=4/\Phi$ is the scattering length due to interface density $\Phi =6f/L_{B,\;I}$ and $l_{q}=1-s_{q}$. In the case of the semi-ab initio thermal conductivity calculations performed in this study, the incorporation of these effective lengths into the overall scattering rate is discussed in Appendix B, specifically Equation (A10).

Next, we discuss the thermal boundary resistance. This is a challenging property to calculate with any accuracy, since the assumptions of the prevailing models are strictly valid only in the continuum limit and exclude the effects of inelastic scattering and other anharmonic processes (see Ref. [25] and citations within), and at best only give plausible bounds on the experimental value of the boundary resistance [4,25]. Nevertheless, we must qualitatively account for the effects of specularity on the thermal boundary resistance in some fashion, and so we follow in the spirit of previous theoretical work on mEMA-type calculations [11] by making use of an approximate model based on Chen’s expressions for the case of superlattice systems [26]. Our intent is to interpolate between the diffuse mismatch model (diffuse limit) and the acoustic mismatch model (specular limit) in a mode-dependent fashion reflecting the value of $s_{q}$, making use of the full band dispersions in our calculation. We use the equivalent equilibrium approach [26], and express $R_{TB}$ as the sum of weighted diffuse $R_{TB}^{D}$ and specular $R_{TB}^{S}$ contributions:

(6) $$R_{TB}=R_{TB}^{D}+R_{TB}^{S}.$$

We begin with $R_{TB}^{D}$. Defining $\langle C_{j}v_{j}\rangle =\sum_{qs}C_{j,\;qs}v_{j,\;qs}$ and ${\langle C_{j}v_{j}\rangle }_{W}=\sum_{qs}C_{j,\;qs}v_{j,\;qs}s_{q}$ where j=i,m denotes the insert or matrix value respectively, $C_{j,\;qs}$ is the specific heat for the mode with momentum q and band number *s* and $v_{j,\;qs}$ is the mode speed (which in the *x* or *y* direction is $\sqrt{v_{x}^{2}+v_{y}^{2}}$ and in the *z* direction is $|v_{z}|$), we may write:

(7) $$R_{TB}^{D}\approx \frac{2({\langle C_{i}v_{i}\rangle }_{W}+{\langle C_{m}v_{m}\rangle }_{W})}{\langle C_{i}v_{i}\rangle \langle C_{m}v_{m}\rangle }.$$

For $R_{TB}^{S}$, having defined the impedences $\langle Z_{j}\rangle =\rho_{j}\sum_{qs}v_{j,\;qs}n_{qs}/\sum_{qs}n_{qs}$ and ${\langle Z_{j}\rangle }_{W}=\rho_{j}\sum_{qs}v_{j,\;qs}l_{q}n_{qs}/\sum_{qs}n_{qs}$ where $\rho_{j}$ is a density and $n_{qs}$ is the Bose-Einstein distribution, we can write the transmission coefficients:

(8) $$\begin{array}{cc} T_{mi}\left(\mu_{i}\right)= & \frac{4\langle Z_{i}\rangle \langle Z_{m}\rangle }{{\left(\langle Z_{i}\rangle \mu_{m}+\langle Z_{m}\rangle \mu_{m}\right)}^{2}},\\ T_{im}\left(\mu_{m}\right)= & \frac{\langle C_{m}v_{m}^{3}\rangle }{\langle C_{i}v_{i}^{3}\rangle }T_{mi}\left(\mu_{i}\right),\\ T_{mi}^{W}\left(\mu_{i}\right)= & \frac{4\langle Z_{i}\rangle \langle Z_{m}\rangle }{{\left({\langle Z_{i}\rangle }_{W}\mu_{i}+{\langle Z_{m}\rangle }_{W}\mu_{m}\right)}^{2}}, \end{array}$$

where $\mu_{j}=\cos\theta_{j}$ where $\theta_{j}$ is the angle of incidence, and $\langle C_{j}v_{j}^{3}\rangle =\sum_{qs}C_{j,\;qs}v_{j,\;qs}^{3}$. Taking $\mu_{c,\;j}$ to be the value of $\mu_{i}$ corresponding to the critical angle above which all incident phonons are reflected, using the following integrals

(9) $$\begin{array}{cc} I_{jk}= & \int_{0}^{\mu_{c,\;j}}T_{jk}\left(\mu_{j}\right)\mu_{j}d\mu_{j},\\ I_{mi}^{W}= & \int_{0}^{\mu_{c,\;m}}T_{mi}^{W}\left(\mu_{m}\right)\mu_{m}d\mu_{m}, \end{array}$$

we may write:

(10) $$R_{TB}^{S}\approx \frac{2}{\langle C_{m}v_{m}\rangle I_{mi}^{W}}\left(1-I_{mi}-I_{im}\right).$$

Note that $R_{TB}$ is not a linearly weighted average, since the weighting of the diffusive contribution enters its definition linearly whereas for the specular contribution it enters quadratically.

As an example calculation, we examine the effects of average inhomogeneity height η on the effective thermal conductivity of bulk 2H WS${}_{2}$ embedded within a matrix of bulk 2H MoS${}_{2}$, where the relevent input thermal conductivities are obtained using a semi-ab initio [24] method described in Appendix B. We assume that the cross-plane and in-plane axes of both materials align, and in calculating the thermal boundary resistances we distinguish the in-plane values $R_{xx}=R_{TB,\;x}=R_{yy}=R_{TB,\;y}$ from the cross-plane values $R_{zz}=R_{TB,\;z}$ by setting $v=\sqrt{v_{x}^{2}+v_{y}^{2}}$ in the former case and $v=|v_{z}|$ in the latter. All other components of R are set to 0.

## 3. Results

**Figure 1:**
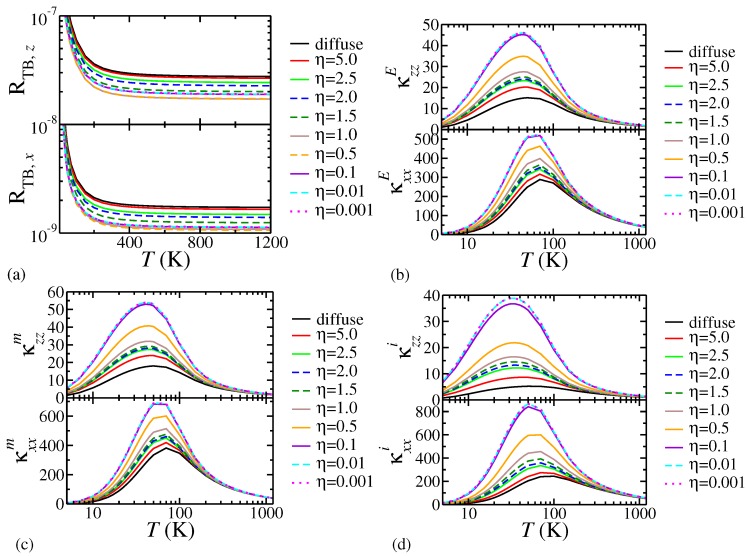
Effect of varying η on (**a**) thermal boundary resistance (note that $R_{TB,\;x}=R_{xx}$ and $R_{TB,\;z}=R_{zz}$); (**b**) effective thermal conductivity; (**c**) matrix thermal conductivity; and (**d**) insert conductivity for bulk 2H WS${}_{2}$ inserts embedded in a matrix of bulk 2H MoS${}_{2}$ with f=0.2, $L_{B,\;I}=1000$ nm, $L_{B,\;S}=10$ μm. The thermal boundary resistance is in units of m${}^{2}$ K W${}^{-1}$, the thermal conductivities are in units of W m${}^{-1}$ K${}^{-1}$.

Figure 1 displays the results varying η in a system with f=0.2, $L_{B,\;S}=10$ μ, and $L_{B,\;I}=1$ μm. Panel (a) displays $R_{TB}$ in the cross-plane (*z*) and in-plane (*x*) directions. In both directions it undergoes a rapid decrease as the temperature rises before saturating above 400 K. $R_{TB}$ at η=5.0 is close to that of the diffuse limit; in the range 0.1≥η≥0.001 we see little change in its value, suggesting that we have attained the specular limit. Any interesting physics regarding the boundary resistance must therefore occur within the range 5.0>η>0.1. We find that $R_{TB,\;z}>R_{TB,\;x}$, which is consistent with $\kappa_{zz}$ being smaller than $\kappa_{xx}$ in the constituent materials. Note that the change in $R_{TB}$ as η decreases is not monotonic: It decreases from the diffuse value, reaches a minimum in the vicinity of η≈1.0 to ≈0.5 and increases towards the specular limiting value. This is a consequence of $s_{q}$ entering into our definition of the diffuse contribution as $s_{q}$ and into the specular contribution as ${\left(1-s_{q}\right)}^{2}$ which would not be seen in a linear mixing approach such as that of Ref. [11].

Panel (b) of Figure 1 displays $\kappa^{E}$ in the cross-plane and in-plane directions. The cross-plane $\kappa_{zz}^{E}$ is, as we would expect from the constituents, smaller than $\kappa_{xx}^{E}$. The overall behaviour with decreasing η in both directions is a monotonic increase from the value in the diffuse limit to saturation as the specular limit is approached for η≤0.1. Note that while η=5.0 does not result in a hugely different value of $R_{TB}$ from that of the diffuse limit, for $\kappa^{E}$ the difference is far more apparent due to the effects of phonon scattering from the interface boundaries.

Results for $\kappa^{m}$ and $\kappa^{i}$ are displayed in panels (c) and (d) of Figure 1, respectively. Similarly to the $\kappa^{E}$ results we see that decreasing η takes us monotonically from the diffuse limit (where scattering from interface boundaries is important) to the specular limit where there is no scattering from interface boundaries, only the sample edges. Interestingly, $\kappa^{m}$ appears to be slightly less sensitive to decreasing η close to the specular limit, becoming saturated at η=0.01 rather than at η=0.1 as is seen for $\kappa^{i}$. This is a result of $L_{IS}$ (the contribution to the scattering length due to interface density) being larger in size than $L_{B,\;I}$; as η tends towards zero the effective scattering length of the matrix will approach the value of the sample length much more quickly.

**Figure 2:**
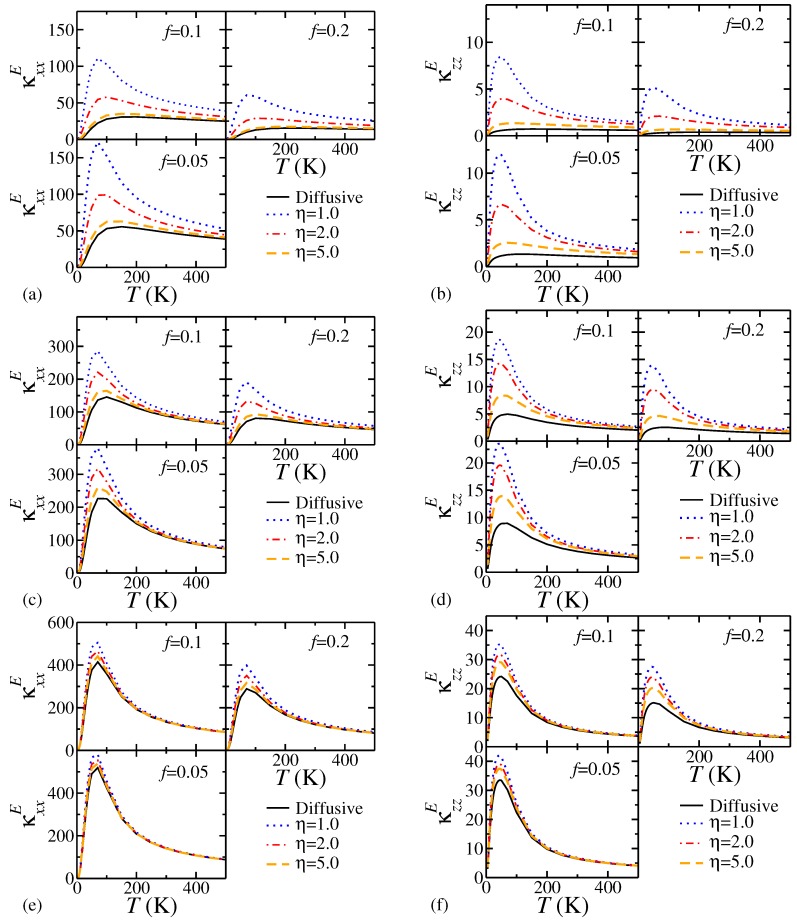
Effects of varying η on the effective thermal conductivities of bulk 2H WS${}_{2}$ inserts embedded in a matrix of bulk 2H MoS${}_{2}$ for $L_{B}=10$ μm and different insert sizes and *f*. (**a**,**b**) $L_{B,\;I}=10$ nm; (**c**,**d**) $L_{B,\;I}=100$ nm; (**e**,**f**) $L_{B,\;I}=1000$ nm. Thermal conductivities are in units of W m${}^{-1}$ K${}^{-1}$.

Figure 2 displays $\kappa_{xx}^{E}$ and $\kappa_{zz}^{E}$ for a number of insert sizes, volume fractions and η values between 5.0 and 1.0, with sample size $L_{B,\;S}=10$ μm. These are intended to capture the effects of physically plausible surface roughnesses, i.e., with deviations from the smooth surface of between 1 and 5 times the lattice constant in height. For all η values, $\kappa^{E}$ in both directions increases with $L_{B,\;I}$ and decreases with *f*, as one would expect. The effect of decreasing η is to reduce the effects of scattering from interfaces, and so we see enhancement of the peak in thermal conductivity below 100 K as the surface of the insert becomes more smooth. This enhancement is most noticable for 10 nm inserts (Figure 2a,b), which in the diffuse limit lack a pronounced peak. As the temperature is increased, the enhancement is decreased, and the thermal conductivities for all finite η values considered tend towards the values seen in the diffuse limit. This is to be expected due to the dominance of anharmonic as opposed to length-based scattering at higher temperatures. The onset of anharmonic dominance generally occurs at lower temperatures for larger insert sizes, however, even at room temperature we can see from Table 1 that the effects of varying η can still be quite significant for 10 nm and 100 nm inserts, particularly for f=0.2.

Why is the deviation from the diffuse result greater for smaller particles? Recall from Equation (5) that the effective boundary length $L_{B}$ for both the insert and the matrix is dependent on both the insert size $L_{B,\;I}$ and $s_{q}$. We may vary $s_{q}$ from 1 (diffuse limit) to 0 (specular limit) by decreasing η. A small $L_{B,\;I}$ means a smaller $L_{B}$ in the diffuse limit than would be the case with a $L_{B,\;I}$, and so for a small $L_{B}$ a wider range of values will be traversed as $s_{q}$ is varied than in the case where it is larger. The same decrease in $s_{q}$ will therefore cause a relatively greater change in $L_{B}$ for the case where $L_{B,\;I}$ is smaller.

For practical purposes, the above conclusion should be qualified slightly. We can estimate from the lattice constant of WS${}_{2}$ that for $L_{B,\;I}=10$ nm, there is a maximum meaningful value of η≈16, which is the point at which the average inhomogeneity height is equal to the radius of the insert. This means that it is entirely possible for η>5.0 and so for calculations considering only diffusive scattering to remain good models. However, if the quality of the interface-matrix boundary is sufficiently high, then the effects of specular scattering from the interfaces of small inserts must be included if the correct effective thermal conductivity is to be calculated.

In fact, comparing these results with those of Ref. [19] we find that the effects of specularity can significantly outweigh the effects of the corrections due to matrix and thermal boundary resistance anisotropy. Behrang et al’s calculation of the effects of specularity through simple mixing for spherical Si inserts in a Ge matrix [11] suggest that only a slight momentum independent specularity is needed to mimic Monte Carlo results. However, direct qualitative comparison between our results and theirs is difficult since they treat thermal boundary resistance differently from the standard approach, such that they predict smaller thermal conductivities than Minnich-Chen type [8] diffuse limit calculations for intermediate *f* and higher thermal conductivities for larger *f*, whereas we would expect a Minnich-Chen calculation (with appropriate modifications as in Ref. [19]) to provide the lower bound for our thermal conductivities.

## 4. Conclusions

We have used an extension of a modified effective medium approach (mEMA) to study thermal conduction in a transition metal dichalcogenide nanocomposite structure with rough interfaces by accounting for the anisotropic conductivities of the host, the insert and the interface regions. The role of specularity in phonon-interface scattering and in thermal boundary resistance is incorporated by implementing a simple phenomenological model relating momentum-dependent specular scattering and surface roughness. In general, it is found that the effect of specular scattering due to interface roughness is more pronounced for inserts smaller than 100 nm in the vicinity of 300 K. In particular, from comparison with calculations carried out in the diffuse limit, for spherical WS${}_{2}$ inserts in a matrix of MoS${}_{2}$, we predict that the effects of specular scattering due to surface roughness is more pronounced for smaller insert sizes, even at volume fractions of the order of 0.05. In other words, the surface roughness of the insert is a key parameter in controlling the thermal conductivity of nanocomposites with insert diameters of the order of tens of nanometers. For applications where the thermal conductivity should be as low as possible (e.g., thermoelectric applications) the surface of such inserts should be as rough as possible.

## Figures and Tables

**Table 1:** Effective thermal conductivities in units of W m${}^{-1}$ K${}^{-1}$ at different η, $L_{B,\;I}$, and volume fraction for $L_{B,\;S}=10$ μm at 300 K.

	Volume Fraction		0.05				0.1				0.2			
		η	Diff.	1.0	2.0	5.0	Diff.	1.0	2.0	5.0	Diff.	1.0	2.0	5.0
	$L_{B,\;I}$													
$\kappa_{xx}^{E}$	**10 nm**		47.9	73.2	59.3	51.7	28.9	52.5	39.8	31.7	15.5	33.7	23.4	16.9
	**100 nm**		110.2	121.9	114.7	112.5	87.8	104.4	94.1	90.8	61.9	80.6	69.2	61.2
	**1000 nm**		140.0	143.5	141.4	140.5	133.0	139.5	135.6	134.0	121.5	132.7	126.0	123.1
$\kappa_{zz}^{E}$	**10 nm**		1.13	2.69	2.28	1.70	0.67	2.15	1.71	1.07	0.37	1.67	1.18	0.61
	**100 nm**		3.81	4.91	4.52	4.37	2.73	4.04	3.64	3.39	1.77	3.17	2.79	2.41
	**1000 nm**		6.41	6.76	6.59	6.56	5.67	6.22	5.97	5.93	4.67	5.44	5.13	5.05

## Abbreviations

The following abbreviations are used in this manuscript:

EMA	Effective Medium Approach
mEMA	modified Effective Medium Approach
emEMA	extended modified Effective Medium Approach
SMRT	Single Mode Relaxation Time
N	Normal anharmonic scattering process
DFT	density functional theory

## Appendix A. Depolarisation Tensors

We first define the affine transformations for the surface and matrix depolarisation tensors as follows [15,17,19]:

(A1) $$\begin{array}{cc} a_{s,\;x} & =\sqrt{\frac{R_{xx}^{K}}{R_{zz}^{K}}}a,\;a_{s,\;y}=\sqrt{\frac{R_{yy}^{K}}{R_{zz}^{K}}}a,\;a_{s,\;z}=a,\\ a_{m,\;x} & =\sqrt{\frac{\kappa_{zz}^{s}}{\kappa_{xx}^{s}}}a,\;a_{m,\;y}=\sqrt{\frac{\kappa_{zz}^{s}}{\kappa_{yy}^{s}}}a,\;a_{m,\;z}=a, \end{array}$$

Since for this system $a_{l,\;z}>a_{l,\;x}=a_{l,\;y}$ for l=s,m and $\kappa^{l}$ is oriented diagonally for its mixing step, we may write the depolarisation tensors as follows [16]:

(A2) $$\begin{array}{cc} N_{l,\;zz} & =\frac{1-e^{2}}{2e^{3}}\left(\ln\frac{1+e}{1-e}-2e\right),\mathrm{where}e=\sqrt{1-\frac{a_{l,\;x}^{2}}{a_{l,\;z}^{2}}},\\ N_{l,\;xx} & =N_{l,\;yy}=\frac{1}{2}\left(1-N_{l,\;zz}\right). \end{array}$$

## Appendix B. Calculation of the Thermal Conductivity

### Appendix B.1. Callaway Thermal Conductivity

Our calculations are performed using the semi-ab initio approach outlined in Ref. [24] with phonon eigensolutions calculated using density functional theory (DFT) as input.

We compute the relaxation time of anharmonic phonon-phonon processes using a mode-averaged, temperature-dependent coupling constant, unlike fully ab initio calculations which numerically estimate third-order force constant tensor elements that are formally valid only at T=0 K. We express the thermal conductivity tensor by solving the linearised phonon Boltzmann equation within a physically appealing generalisation of Callaway’s scheme to obtain an effective relaxation time [27]. Recently, some research groups have adopted an ‘iterative’ approach for solving the Boltzmann equation for an effective relaxation time. Such an approach can be related to the concept of a variational method described in Ref. [28]. An early work by one of the present authors [29] indicates that the resulting conductivites would be similar in magnitude to those of the Callaway scheme, as does a more recent work [30]. More importantly, our overall approach has been validated against experimental measurements of Si and Ge thermal conductivities over a wide temperature range [24]. The present approach is briefly outlined below.

We work within the Boltzman transport approach [28], using a generalization [27] of Callaway’s [31] improvement on the single-mode relaxation time (SMRT) solution which accounts for the momentum conservation of Normal (N) phonon-phonon scattering processes through the inclusion of an additional ‘N-drift’ term. The thermal conductivity tensor is defined as follows [27]:

(A3) $$\begin{array}{ccc} \kappa_{ij} & = & \frac{\hbar^{2}}{N_{0}\Omega k_{B}T^{2}}[\sum_{qs}v_{s}^{i}\left(q\right)v_{s}^{j}\left(q\right)\omega^{2}\left(qs\right)\tau_{qs}\bar{n}\left(qs\right)\left(\bar{n}\left(qs\right)+1\right)\\ & & +A_{C}\sum_{qs}q^{i}v_{s}^{j}\omega \left(qs\right)\tau_{qs}\tau_{qs,N}^{-1}\bar{n}\left(qs\right)\left(\bar{n}\left(qs\right)+1\right)], \end{array}$$

(A4) $$\begin{array}{cc} = & \kappa_{ij}^{\mathrm{SMRT}}+\kappa_{ij}^{N-\mathrm{drift}}. \end{array}$$

Here, $A_{C}$ is temperature dependent but polarisation and phonon wave-vector independent, and is expressed as:

(A5) $$A_{C}=\frac{\sum_{qs}q^{i}v_{s}^{j}\omega \left(qs\right)\tau_{qs}\tau_{qs,N}^{-1}\bar{n}\left(qs\right)\left(\bar{n}\left(qs\right)+1\right)}{\sum_{qs}q^{i}q^{j}\tau_{qs,N}^{-1}\left(1-\tau_{qs}\tau_{qs,N}^{-1}\right)\bar{n}\left(qs\right)\left(\bar{n}\left(qs\right)+1\right)}.$$

In the above, Ω is the unit cell volume and $N_{0}$ their number; ω(qs) is the frequency of a phonon with wavevector q and polarisation *s*; $v_{s}^{i}\left(q\right)$ is the corresponding group velocity component; $\bar{n}\left(qs\right)$ is the Bose-Einstein distribution function; $\tau_{qs}$ is the SMRT relaxation time; and $\tau_{qs,N}$ is the contribution to $\tau_{qs}$ from N-type phonon-phonon anharmonic scattering processes. (We note in passing that the above is a correction to the expressions given in Refs. [24,32], which erroniously state a form valid only for linear phonon dispersions as opposed to the general form [27] that is actually used in our calculations.)

Contributions to the SMRT are summed as follows:

(A6) $$\begin{array}{cc} \tau_{qs}^{-1} & =\tau_{qs}^{-1}\left(\mathrm{bulk}\right)+\tau_{qs}^{-1}\left(\mathrm{IF}/B\right),\\ \tau_{qs}^{-1}\left(\mathrm{bulk}\right) & =\tau_{qs}^{-1}\left(\mathrm{md}\right)+\tau_{qs,N}^{-1}+\tau_{qs,U}^{-1}. \end{array}$$

Here $\tau_{qs}^{-1}\left(\mathrm{bulk}\right)$ is the total scattering rate for a bulk system, which is the sum of the mass-defect and anharmonic (both Normal and Umklapp) scattering rates. $\tau_{qs}^{-1}\left(\mathrm{IF}/B\right)$ is the contribution from interface or boundary scattering.

### Appendix B.2. Bulk Scattering Rates

The isotopic mass defect scattering rate is given by [28]:

(A7) $$\tau_{qs}^{-1}\left(\mathrm{md}\right)=\frac{\pi }{2N_{0}}\omega_{qs}^{2}\sum_{q^{\prime }s^{\prime }}\delta \left(\omega_{qs}-\omega_{q^{\prime }s^{\prime }}\right)\sum_{b}\Gamma_{\mathrm{md}}\left(b\right){\left|e_{qs}^{٭}\left(b\right)\cdot e_{q^{\prime }s^{\prime }}\left(b\right)\right|}^{2},$$

where $e_{qs}\left(b\right)$ is an eigenvector of the lattice dynamical matrix and b labels an atomic site within the unit cell. For a given atom the mass-disorder coefficient is:

(A8) $$\Gamma_{\mathrm{md}}\left(b\right)=\sum_{i}f_{i}\left(b\right){\left[1-M_{i}\left(b\right)/\bar{M}\left(b\right)\right]}^{2},$$

where $f_{i}\left(b\right)$ is the frequency of isotope *i* of the atomic species at b, $M_{i}\left(b\right)$ the corresponding isotope mass and $\bar{M}\left(b\right)$ is the average mass of that atomic species.

The semi-ab initio approach [24] is used to calculate the three phonon anharmonic contributions [28]:

(A9) $$\begin{array}{ccc} \tau_{3\mathrm{ph},\;qs}^{-1} & = & \frac{\pi \hbar }{\varrho N_{0}\Omega }\frac{\bar{\gamma^{2}}\left(T\right)}{{\bar{c}}^{2}}\sum_{q^{\prime }s^{\prime },\;q^{\prime \prime }s^{\prime \prime },\;G}\omega \omega^{\prime }\omega^{\prime \prime }\delta_{q+q^{\prime }+q^{\prime \prime },G}\\ & \times & \left[\frac{{\bar{n}}^{\prime }\left({\bar{n}}^{\prime \prime }+1\right)}{\left(\bar{n}+1\right)}\delta \left(\omega +\omega^{\prime }-\omega^{\prime \prime }\right)+\frac{1}{2}\frac{{\bar{n}}^{\prime }{\bar{n}}^{\prime \prime }}{\bar{n}}\delta \left(\omega -\omega^{\prime }-\omega^{\prime \prime }\right)\right], \end{array}$$

where ϱ is the mass density, $\bar{c}$ is the average acoustic velocity and we have suppressed the wavevector and polarisation indices for ω and $\bar{n}$ for reasons of brevity. The mode-averaged, temperature dependent squared Grüneisen parameter $\bar{\gamma^{2}}\left(T\right)$ is calculated using the quasi-harmonic approximation [28,33,34,35]. If G=0 then the process is a Normal one, otherwise it is an Umklapp process.

### Appendix B.3. Sample Boundary and Interface Scattering Rates

These enter into the total scattering time as:

(A10) $$\tau_{qs}^{-1}\left(\mathrm{IF}/B\right)=\frac{|v_{s}\left(q\right)|}{L_{B}},$$

where $L_{B}$ for the matrix and insert are defined in Equation (5).

### Appendix B.4. Computational Details

Full details of the generation of phonon eigensolutions for MoS${}_{2}$ and WS${}_{2}$ are given in [32]. Calculations used the Quantum Espresso DFT package [36] with PBE [37] pseudopotentials on 8×8×2 Monkhorst-Pack (MP) grids [38]. From these force constants we obtained the frequencies, eigenvectors and velocities needed for thermal conductivity calculations using 28×28×7 MP grids.
